# Aurora-A Mitotic Kinase Induces Endocrine Resistance through Down-Regulation of ERα Expression in Initially ERα+ Breast Cancer Cells

**DOI:** 10.1371/journal.pone.0096995

**Published:** 2014-05-09

**Authors:** Mateusz Opyrchal, Jeffrey L. Salisbury, Shuya Zhang, James McCubrey, John Hawse, Mattew P. Goetz, Gwen A. Lomberk, Tufia Haddad, Amy Degnim, Carol Lange, James N. Ingle, Evanthia Galanis, Antonino B. D'Assoro

**Affiliations:** 1 Department of Medical Oncology, Mayo Clinic College Of Medicine, Rochester, Minnesota, United States of America; 2 Department of Biochemistry and Molecular Biology, Mayo Clinic College Of Medicine, Rochester, Minnesota, United States of America; 3 Department of Molecular Medicine, Mayo Clinic College Of Medicine, Rochester, Minnesota, United States of America; 4 Department of General Surgery, Mayo Clinic College Of Medicine, Rochester, Minnesota, United States of America; 5 Department of Microbiology and Immunology, East Carolina University, Greenville, North Carolina, United States of America; 6 Departments of Medicine and Pharmacology, University of Minnesota, Minneapolis, Minnesota, United States of America; Institut de Génomique Fonctionnelle de Lyon, France

## Abstract

Development of endocrine resistance during tumor progression represents a major challenge in the management of estrogen receptor alpha (ERα) positive breast tumors and is an area under intense investigation. Although the underlying mechanisms are still poorly understood, many studies point towards the ‘cross-talk’ between ERα and MAPK signaling pathways as a key oncogenic axis responsible for the development of estrogen-independent growth of breast cancer cells that are initially ERα+ and hormone sensitive. In this study we employed a metastatic breast cancer xenograft model harboring constitutive activation of Raf-1 oncogenic signaling to investigate the mechanistic linkage between aberrant MAPK activity and development of endocrine resistance through abrogation of the ERα signaling axis. We demonstrate for the first time the causal role of the Aurora-A mitotic kinase in the development of endocrine resistance through activation of SMAD5 nuclear signaling and down-regulation of ERα expression in initially ERα+ breast cancer cells. This contribution is highly significant for the treatment of endocrine refractory breast carcinomas, because it may lead to the development of novel molecular therapies targeting the Aurora-A/SMAD5 oncogenic axis. We postulate such therapy to result in the selective eradication of endocrine resistant ERα^low/−^ cancer cells from the bulk tumor with consequent benefits for breast cancer patients.

## Introduction

Approximately, 70% of human breast carcinomas fall into luminal subtypes, which are estrogen receptor alpha (ERα) positive [1]. ERα expression correlates with expression of progesterone receptor (PR), lower tumor grade, response to endocrine therapy, lower grade of aneuploidy, less frequent overexpression of HER-2 oncogene, bone metastases and slower rate of tumor recurrence [2]. Despite the clinical benefit of hormonal treatment in patients with ERα+ breast cancer, resistance to first and second-line endocrine therapy remains a major clinical problem [3], [4]. The introduction of pure estrogen antagonists such as fulvestrant, to overcome the apparent disadvantage of tamoxifen with its partial agonist properties, did not resolve the endocrine resistance problem [5]. Second-line therapy with other endocrine agents such as aromatase inhibitors produces some beneficial effect but for the most part serves merely to delay onset of endocrine resistance [6]. In pre-clinical and clinical studies, development of endocrine resistance is associated with an aggressive behavior characterized by high frequency of distant metastases and poses a significant problem that affects negatively the disease-free and overall survival of breast cancer patients [7]. Response to one form of endocrine therapy after resistance to a previous therapy is a historically recognized observation that is the key to management of patients with metastatic disease [8]. Importantly, subsequent responses to serial endocrine therapy tend to be shorter, indicating a gradual shift from “*dependence on ERα signaling to alternative escape oncogenic pathways*” [9]. Several mechanisms of endocrine resistance have been proposed including: ERα mutations, altered expression of ERα coregulators, ligand-independent activation of ERα by growth factor receptor kinases and down-regulation/loss of ERα expression [3]. Importantly, whereas “*acquired resistance*” is predominantly the consequence of estrogen-independent activation of ERα in cancer cells still harboring an intact ERα signaling axis, “*intrinsic resistance*” is mostly likely due to down-regulation/loss of ERα expression leading to endocrine panresistance and tumor progression and represents a serious challenge for the treatment of ERα+ breast cancer patients [3]. For this reason, understanding the oncogenic pathways responsible for the development of resistance to endocrine therapy given before or after primary surgery is imperative to develop innovative therapeutic strategies aimed to suppress or at least delay recurrence and progression of initially ERα+ breast tumors.

The discovery that breast carcinomas contain a sub-population of cells harboring stem-like properties (cancer initiating cells) has generated excitement because these cancer initiating cells may represent the source of therapeutic failures, tumor recurrence and poor clinical outcome [10]. It has also been demonstrated that cancer cells that undergo through epithelial to mesenchymal transition (EMT) acquire a basal CD44^+^/CD24^low/−^ cancer stem-like phenotype with increased capacity for self-renewal, invasion, drug resistance, and tumor progression [11]. Moreover, breast cancer initiating cells display down-regulation of ERα and clonal expansion of these CD44^+^/CD24^low/−^/ERα^low/−^ cancer cells may be responsible for tumor recurrence and development of distant metastases of initially ERα+ breast carcinomas [12], [13]. Aberrant activation of HER-2/MAPK and TGFβ/SMAD oncogenic signalings induces EMT and plays an important role in the maintenance of breast cancer initiating cells [14]–[16]. However, the underlying molecular mechanisms responsible for ERα down-regulation by aberrant activation of HER-2/MAPK and TGFβ/SMAD pathways remain elusive and are the subject of ongoing investigation.

The mitotic kinase Aurora-A plays a key role in breast cancer progression through the development of centrosome amplification and chromosomal instability (CIN) [17]–[19]. Aurora-A is over-expressed in human breast tumors and is associated with an invasive ERα^low/-^ basal-like phenotype and poor-prognosis [20], [21]. Moreover, it has been demonstrated that estrogen is causally linked via ERα to Aurora-A overexpression, centrosome amplification, CIN, and aneuploidy leading to breast tumors in susceptible mammary gland cells [22]. Nonetheless, the causal role of aberrant Aurora-A kinase activity in the development of endocrine resistance and breast cancer progression through molecular mechanisms that are independent from its mitotic function and CIN remains elusive. Herein we demonstrate a significant and novel non-mitotic role of Aurora-A kinase in the induction of tumor progression of ERα+ breast cancer xenografts through activation of EMT and the genesis of CD44^+^/CD24^low/−^ cancer initiating cells [23]. Moreover, these studies revealed a non canonical cross-talk between Aurora-A kinase and SMAD5 oncogenic signaling in promoting EMT and invasiveness. We demonstrate for the first time the causal role of Aurora-A/SMAD5 oncogenic axis in the development of endocrine resistance through down-regulation of ERα expression in initially ERα+ breast cancer cells. This contribution is important for the treatment of endocrine refractory breast carcinomas, because it may lead to the development of novel molecular therapies targeting the Aurora-A/SMAD5 oncogenic axis. We postulate such therapy to result in selective eradication of endocrine resistant ERα^low/−^ cancer cells from the bulk tumor thereby delaying tumor progression with consequent benefits on the progression-free and overall survival of breast cancer patients.

## Results and Discussion

**Figure 1:**
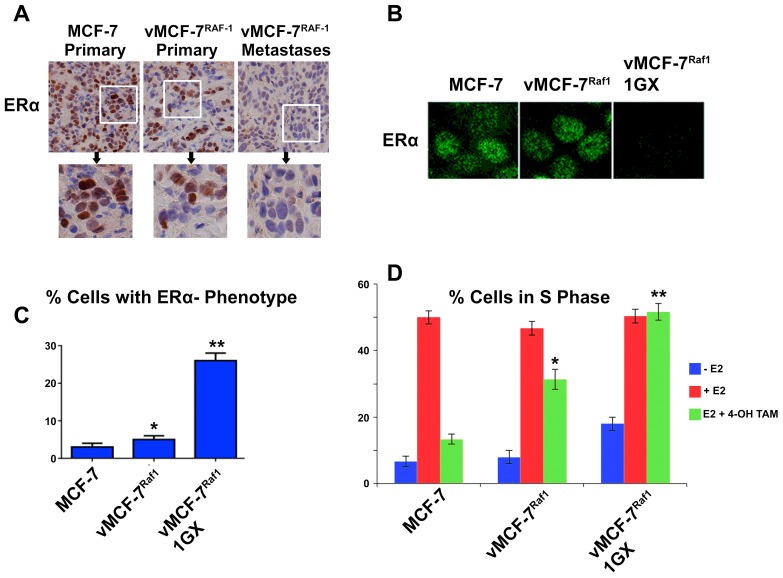
Endocrine Resistant Breast Cancer Cells. (**A**) Immumohistochemistry staining of low-grade tubular tumors for MCF-7 and high-grade vMCF-7^ΔRaf-1^ Primary and Metastatic tumors. Breast cancer xenografts were stained with a polyclonal antibody targeting the ERα (Abcam, Cambridge, Massachusetts, USA). (**B**) Immunofluorescence analysis showing down-regulation of ERα expression in vMCF-7^ΔRaf-1^ 1GX cancer cells compared to parental MCF-7 and vMCF-7^ΔRaf-1^ cells. (**C**) Graph showing the percentage of cancer cells harboring an ERα^low/−^ phenotype from three independent experiments (+/− s.d.; *p<0.0705 vs. MCF-7; **p<0.0001 vs. vMCF-7^ΔRaf-1^). (**D**) Graph showing the percentage of cancer cells in the S phase of the cell cycle during starvation from 17-β estradiol and following treatment with 17-β estradiol (10^−10^ M) alone or in combination with 4-OH-tamoxifen (10^−7^ M) for 48 hours from three independent experiments (+/− s.d.; *p<0.0008 vs. MCF-7; **p<0.0009 vs. vMCF-7^ΔRaf-1^).

**Figure 2:**
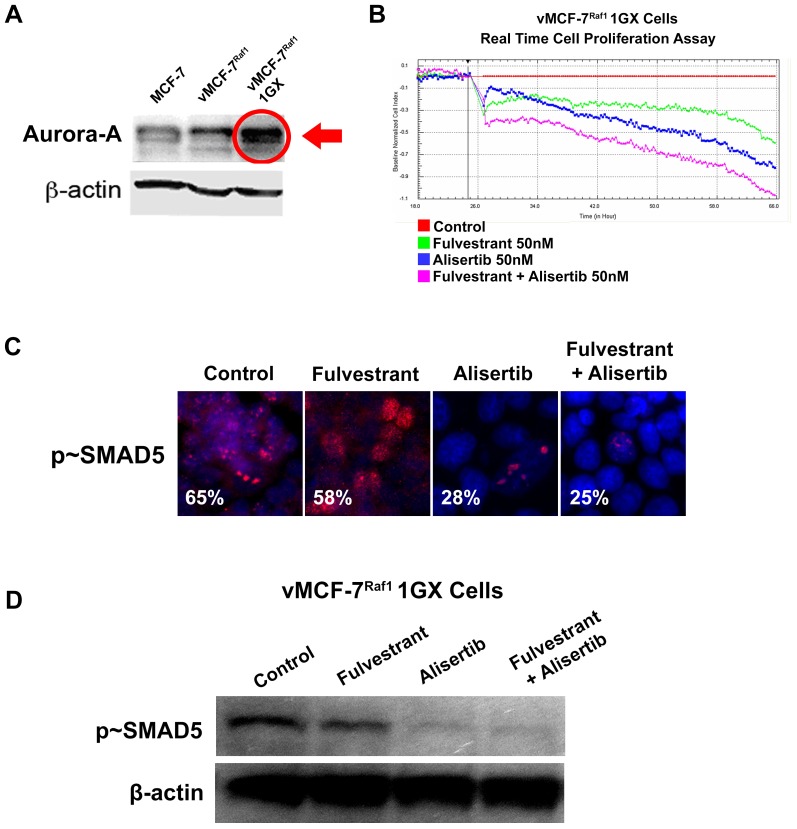
Molecular characterization of endocrine resistant breast cancer cells. (**A**) Immunoblot analysis showing Aurora-A (Cell Signaling Technology, Boston, MA, USA) over-expression in vMCF-7^ΔRaf-1^ 1GX cancer cells compared to parental MCF-7 and vMCF-7^ΔRaf-1^ cells. (**B**) *In Vitro* Real Time Cell Proliferation Assay showing stronger activity of Fulvestrant (50 nM) in combination with alisertib (50 nM) in tamoxifen resistant vMCF-7^ΔRaf-1^ 1GX cancer cells. Experiments were performed in triplicate. (**C**) Immunofluorescence analysis showing inhibition of nuclear SMAD5 (Cell Signaling Technology, Boston, MA, USA) phosphorylation in breast cancer cells treated with alisertib. p∼SMAD5 was labeled in red and nuclei were labeled in blue with DAPI. (**D**) Immunoblot analysis showing selective alisertib-induced down-regulation of SMAD5 phosphorylation.

**Figure 3:**
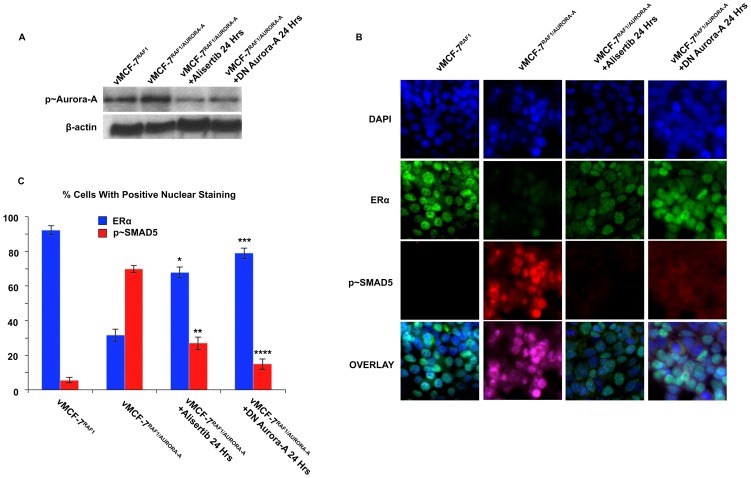
Mechanistic Linkage Between Aurora-A Over-expression, SMAD5 Activation And ERα Down-Regulation In Initially ERα+ Breast Cancer Cells. (A) Immunoblot analysis showing p∼Aurora-A in breast cancer cells. (B) Immunofluorescence analysis showing that Aurora-A-induced ERα down-regulation is linked to p∼SMAD5 nuclear activation. ERα (Abcam, Cambridge, Massachusetts, USA) was labeled in green, p∼SMAD5 (Cell Signaling Technology, Boston, MA, USA) was labeled in red and nuclei were labeled in blue with DAPI. (C) Graph showing the percentage of cells expressing ERα and p∼SMAD5 in breast cancer cells. Experiments were performed in triplicate (+/− s.d.; *p<0.0083 vs. vMCF-7^ΔRaf-1/Aurora-A^; **p<0.0048 vs. vMCF-7^ΔRaf-1/Aurora-A^; ***p<0.0083 vs. vMCF-7^ΔRaf-1/Aurora-A^; ****p<0.0021 vs. vMCF-7^ΔRaf-1/Aurora-A^).

**Figure 4:**
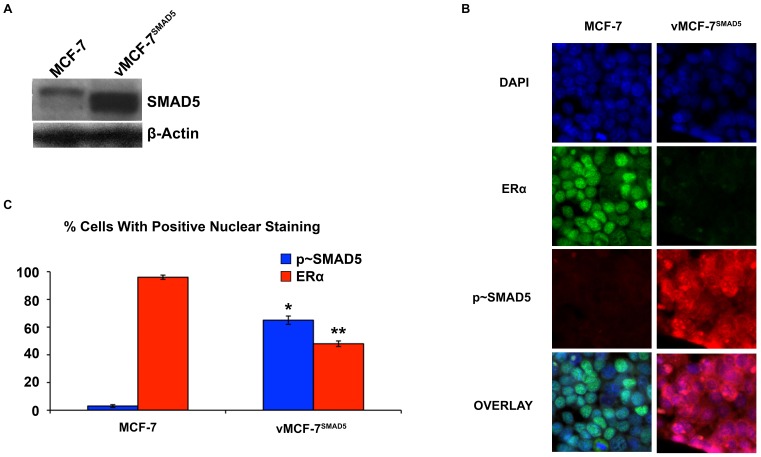
Role Of SMAD5 Over-Expression In ERα Down-Regulation. (**A**) Immunoblot analysis showing parental and MCF-7 cells engineered to over-express SMAD5. (**B**) Immunofluorescence analysis showing that SMAD5 over-expression induces ERα down-regulation in ERα+MCF-7 cells. ERα (Abcam, Cambridge, Massachusetts, USA) was labeled in green, p∼SMAD5 (Cell Signaling Technology, Boston, MA, USA) was labeled in red and nuclei were labeled in blue with DAPI. (**C**) Graph showing the percentage of cells expressing p∼SMAD5 and ERα in vMCF-7^SMAD5^ and parental cells. Experiments were performed in triplicate (+/− s.d.; *p<0.0001 vs. MCF-7; **p<0.0001 vs. MCF-7).

Because development of endocrine resistance and progression of ERα+ breast tumors is frequently characterized by aberrant activation of MAPK signaling [24], we employed ERα+ MCF-7 cells over-expressing a constitutive active Raf-1 oncoprotein (vMCF-7^ΔRaf-1^) as previously described [23], [25]. In our previous studies we have showed that vMCF-7^ΔRaf-1^ cells display MAPK hyper-phosphorylation compared to parental MCF-7 cells, demonstrating a constitutive activation of Raf/MAPK oncogenic signaling. Importantly, constitutive activation of Raf/MAPK signaling conferred higher migratory properties of MCF-7 cells *in vitro*, predictive of an invasive phenotype that was validated *in vivo* through the development of distant metastases in tumor xenograft models. To investigate the extent to which metastatic lesions derived from vMCF-7^ΔRaf-1^ xenografts displayed ERα down-regulation, we established murine MCF-7 and vMCF-7^ΔRaf-1^ xenografts. Tumor xenografts were surgically removed 12 weeks after implantation without sacrificing the animals to monitor the development of distant metastases as previously described [25]. As expected, 8 weeks following surgical removal, only vMCF-7^ΔRaf-1^ xenografts developed frank distant metastases (lung and spleen). Importantly, vMCF-7^ΔRaf-1^ metastatic lesions showed ERα down-regulation resulting in ERα^+/−^ cell heterogeneity compared to MCF-7 and vMCF-7^ΔRaf-1^ primary tumors (Figure 1A). These findings indicate that ERα^low/−^ cancer cells display more invasive properties over ERα+ cancer cells *in vivo* and their clonal expansion may induce tumor progression. To investigate whether vMCF-7^ΔRaf-1^ primary tumors carried a singular sub-population of cancer cells harboring an ERα^low/-^ phenotype that was mostly observed in the metastatic lesions described above, we re-cultured cells from primary vMCF-7^ΔRaf-1^ tumor xenografts (referred to as first generation derived from xenografts, 1GX). Significantly, vMCF-7^ΔRaf-1^ 1GX cells showed down-regulation of ERα expression due to loss of ERα in ∼28% of bulk cancer cells (Figure 1B–C). These findings demonstrate that cancer cells harboring an ERα^low/−^ phenotype were already present in vMCF-7^ΔRaf-1^ primary tumors and their clonal expansion may promote the onset of distant metastases during tumor progression. Next we investigated whether down-regulation of ERα expression was causally linked to development of endocrine resistance in vMCF-7^ΔRaf-1^ 1GX cells. Parental MCF-7 and variant cells were treated *in vitro* with 17-β Estradiol alone or in combination with the anti-estrogen 4-OH-tamoxifen and endocrine sensitivity was determined by analyzing the percentage of cancer cells in the S phase of the cell cycle. vMCF-7^ΔRaf-1^ 1GX cells displayed the highest resistance to 4-OH tamoxifen compared to parental MCF-7 and vMCF-7^ΔRaf-1^ cells indicating that down-regulation of ERα induces “*intrinsic resistance*” to conventional endocrine therapy (Figure 1D). Since we have previously shown that tumor progression of vMCF-7^ΔRaf-1^ 1GX xenografts is causally linked to aberrant Aurora-A kinase activity [23], we tested Aurora-A expression in MCF-7 and variant cells. As demonstrated before, Aurora-A was over-expressed in vMCF-7^ΔRaf-1^ 1GX cells compared to parental cells (Figure 2A). Next we investigated the causal role of Aurora-A kinase activity in the development of endocrine resistance by employing alisertib, a novel Aurora-A kinase small molecule inhibitor currently being tested in oncology clinical trials [26]. We have previously demonstrated that treatment of breast cancer cells with 1 µM alisertib selectively inhibits Aurora-A kinase activity [23]. Specifically, our study showed that the transcriptome profile of breast cancer cells treated with alisertib or shRNA targeting Aurora-A displayed ∼90% gene expression overlapping demonstrating the high specificity of 1 µM alisertib for targeting Aurora-A kinase activity. vMCF-7^Raf1^ 1GX cells that displayed strong 4-OH tamoxifen resistance were treated with fulvestrant (a selective ERα down-regulator that increases ERα degradation and inhibits estrogen signaling) alone or in combination with alisertib. Combination of fulvestrant with alisertib induced a stronger effect on inhibition of cell proliferation measured by Real-Time Cell Proliferation Assay (Figure 2B). Based on our previous results viewing a novel cross-talk between Aurora-A kinase and SMAD5 oncogenic signaling in the development of EMT and breast cancer progression [23], we also analyzed SMAD5 nuclear phosphorylation in vMCF-7^Raf1^ 1GX cells treated with fulvestrant and/or alisertib. Importantly, restoration of endocrine sensitivity was mechanistically linked to suppression of SMAD5 nuclear phosphorylation (Figure 2C), demonstrating the causal role of Aurora-A/SMAD5 oncogenic axis in the development of endocrine resistance in initially ERα+ and hormone sensitive breast cancer cells. Significantly, these findings were also validated by performing immunoblotting analysis of vMCF-7^Raf1^ 1GX cells treated with fulvestrant and/or alisertib showing a selective alisertib-induced down-regulation of SMAD5 phosphorylation (Figure 2D). Next we wanted to investigate the causal role of Aurora-A/SMAD5 oncogenic axis in the development of “*intrinsic resistance*” to conventional endocrine therapy through down-regulation of ERα expression in initially ERα+vMCF-7^Raf1^ cancer cells. We employed a lenti-vector engineered to over-express Aurora-A kinase and we analyzed Aurora-A phosphorylation/activation, ERα and p∼SMAD5 nuclear localization in vMCF-7^Raf-1^ and vMCF-7^Raf-1/Aurora-A^ cells (Figure 3A–B). Our studies showed that aberrant Aurora-A kinase activity induced down-regulation of ERα nuclear localization that was functionally linked to increased SMAD5 nuclear phosphorylation (Figure 3B–C). Importantly, treatment of vMCF-7^Raf-1/Aurora-A^ cells with alisertib inhibited Aurora-A phosphorylation, restored ERα nuclear expression and suppressed SMAD5 nuclear phosphorylation (Figure 3B–C). Moreover, the causal role of Aurora-A kinase in the down-regulation of ERα nuclear expression through activation of SMAD5 was validated by employing a dominant negative (DN) Aurora-A construct that abrogated Aurora-A kinase activity (Figure 3B–C). Significantly, over-expression of Aurora-A in parental MCF-7 cells resulted in a similar phenotype, although vMCF-7^Raf-1/Aurora-A^ cells displayed a stronger phosphorylation of nuclear SMAD5 and down-regulation of ERα expression, demonstrating a synergistic cross-talk between MAPK pathway and aberrant Aurora-A kinase activity in the abrogation of ERα signaling (Figure S1). Finally, to investigate the role of SMAD5 as a down-stream target of Aurora-A-induced ERα down-regulation, we engineered MCF-7 over-expressing SMAD5 (Figure 4A). Notably, over-expression of SMAD5 in MCF-7 cells induced down-regulation of ERα nuclear localization demonstrating the causal role of SMAD5 nuclear signaling in the development of breast cancer cells harboring an ERα^low/−^ phenotype (Figure 4B–C). Taken together, these results indicate that the partial response of endocrine resistant vMCF-7^Raf1^1GX cancer cells to fulvestrant is likely due to its effect on the ERα+ sub-population that still retains a functional ERα signaling. Inhibition of Aurora-A kinase activity by alisertib impairs SMAD5 nuclear activation and restores ERα expression in the subpopulation of ERα^low/−^ cancer cells leading to restoration of endocrine sensitivity. Furthermore, our findings are supported by recent studies that have demonstrated that down-regulation of ERα expression is responsible for endocrine therapeutic failure and tumor progression of initially ERα+ breast tumors [27]. Although it has already been demonstrated that key transcription factors involved in the development of EMT such as Snail and Slug induce down-regulation of ERα expression in breast cancer cells [28], [29], our findings establish a previously unrecognized oncogenic signaling involved in the abrogation of ERα function through activation of the Aurora-A/SMAD5 oncogenic axis. Moreover, vMCF-7^ΔRaf-1^ breast cancer xenografts represent an innovative and useful pre-clinical model to investigate the mechanisms leading to endocrine panresistance and tumor progression based on the clonal expansion of ERα^low/−^ cancer cells. Importantly, although these studies are based on the vMCF-7^ΔRaf-1^ xenograft model derived from one single cell line, global gene expression analysis have shown that vMCF-7^Raf1^ 1GX cancer cells developed a distinct transcriptome signature characterized by activation of EMT and stemness signalings compared to parental MCF-7 cells [23].

**Figure 5:**
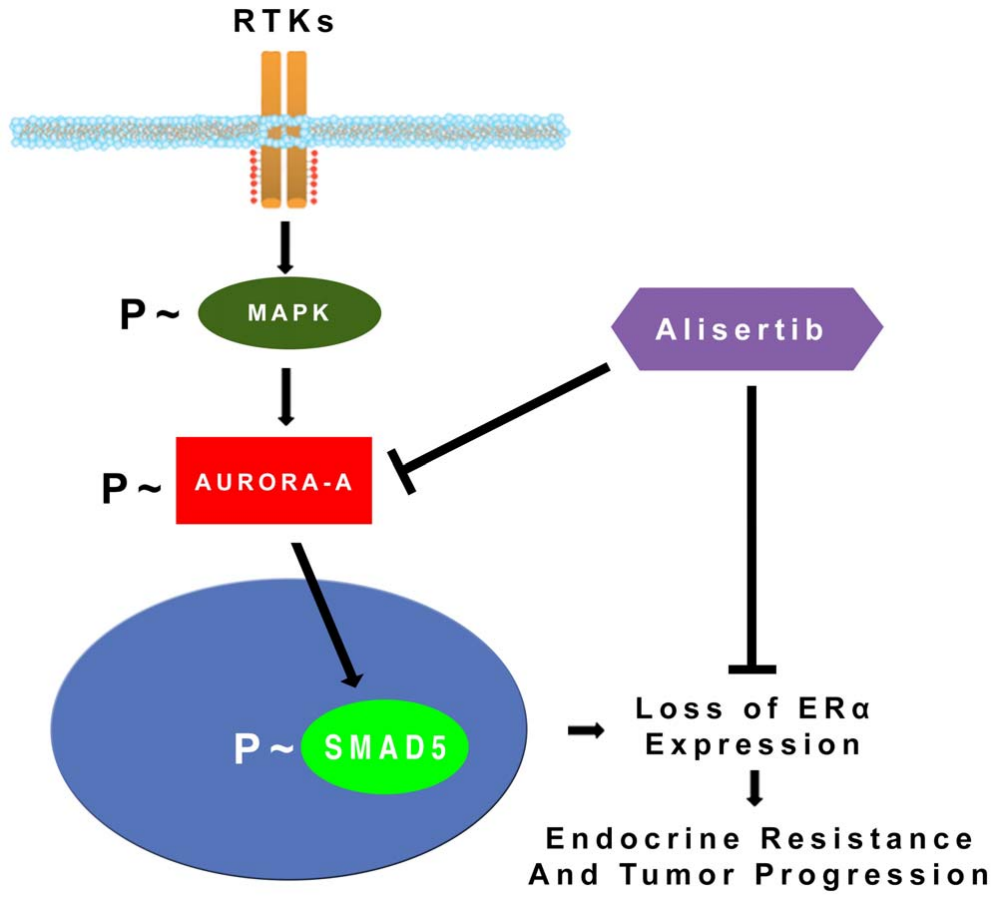
Model of endocrine resistance and breast cancer progression. Aberrant activation of MAPK signaling stabilizes and activates Aurora-A kinase that in turn induces down-regulation/loss of ERα expression through phosphorylation and activation of SMAD5 nuclear signaling leading to endocrine resistance and tumor progression.

Based on these findings and our published data [23], [25], we propose a novel model of endocrine resistance in initially ERα+ and hormone sensitive breast cancer cells: aberrant activation of MAPK signaling promotes phosphorylation and stabilization of Aurora-A kinase that in turn induces down-regulation/loss of ERα expression through activation of SMAD5 nuclear signaling leading to endocrine resistance and tumor progression (Figure 5). Because our previous studies have demonstrated the causal role of aberrant Aurora-A kinase activity in the development of CD44^+^/CD24^low/−^ cancer initiating cells [23], we speculate that Aurora-A/SMAD5 oncogenic axis may induce ERα down-regulation by promoting the clonal expansion of CD44^+^/CD24^low/-^ cancer initiating cells that display low levels of ERα. Moreover, because Aurora-A is down-stream of MAPK, we believe that molecular targeting of Aurora-A in endocrine resistant breast cancer cells will be more effective than targeting MAPK pathway due to Aurora-A direct effects on the clonal expansion of CD44^+^/CD24^low/−^/ERα^low/−^ cancer initiating cells [23]. However, we don't exclude that patients with endocrine resistant breast tumors could benefit from the combination of MAPK and Aurora-A small molecule inhibitors.

In conclusion, our studies are of important translational relevance because they will lay the groundwork for innovative clinical trials employing small molecule inhibitors of Aurora-A kinase to suppress the Aurora-A/SMAD5 oncogenic axis, restore ERα expression and sensitivity to endocrine therapy for a subset of hormone refractory breast tumors with anticipated benefits on the progression-free and overall survival of breast cancer patients.

## Materials and Methods

### Human Breast Cancer Cell Lines

The human breast cancer cell line MCF-7 was obtained from ATCC (Manassas, VA, USA). The MCF-7 cells over-expressing the Raf-1 oncoprotein were generated as previously described [23], [25]. All cell lines were maintained in EMEM medium containing 5 mM glutamine, 1% penicillin/streptomycin, 20 microgram insulin/ml and 10% FBS at 37C in 5% CO2 atmosphere.

### Tumor Xenografts and Immunohistochemistry

Procedures established by the Institutional Animal Care and Use Committee based on US NIH guidelines for the care and use of laboratory animals were followed for all experiments. Four-week-old non-ovariectomized female NCR/Nu/Nu nude mice were anesthetized by exposure to 3% isoflurane and five mice per each group were injected subcutaneously with 2×10^6^ MCF-7 or vMCF-7^ΔRaf-1^ cancer cells suspended in 50 µl of 50% Matrigel (BD Bioscience, Bedford, MA, USA). Tumor localization and growth was monitored using the IVS imaging system from the ventral view 10 min after luciferin injection. After 12 weeks, mice were killed and xenograft tumors were processed for histology, and immunohistochemistry analyses. Paraffin-embedded tumor tissues were stained with ERα antibody (Abcam, Cambridge, Massachusetts, USA) as previously described [23]. To re-establish cultures from 1GX explants, primary tumors tissues were excised from killed animals, minced using sterile scissors, transferred to complete culture medium and fibroblast-free tumor cell lines were established by serial passages in culture. Animals were examined everyday and body weight and primary tumor size were measured at least 1–2 times per week. Consistent distress and potential pain (>1 day) were alleviated by euthanasia. If some of the animals were loosing greater than 10% of their body weight, if blood was consistently observed in the urine or around the genitals of the mice, the mice were appropriately euthanized. When typical signs of distress including labored breathing and inactivity were consistently observed for >1 day, the animals were appropriately euthanized. When the primary tumor was >2 cm, the animals were sacrificed. Animals were euthanized using Pentobarbital (IP 100 mg/kg) followed by cervical dislocation. The Mayo Clinic Institutional Animal Care and Use Committee (IACUC) approved this study.

### Fluorescence Microscopy

Cells were fixed in absolute methanol at −20°C for 10 min, blocked in 5% normal goat serum, 1% glycerol, 0.1% BSA, 0.1% fish skin gelatin, 0.04% sodium azide and incubated with primary antibodies. Primary antibodies against the proteins ERα (Santa Cruz Biotechnology, Delaware Avenue Santa Cruz, CA, USA) and P∼SMAD5 (Cell Signaling Technology, Boston, MA, USA) were followed by secondary antibodies conjugated with Alexa 488 or Alexa 568 (Molecular Probes, Eugene, OR, USA). Images were digitally recorded at multiple focal planes using a Zeiss Axiovert 200 M fluorescence microscope and analyzed as maximum projections. Results are derived from three independent experiments. Nuclear staining counting was performed employing the *ImageJ Software*, experiments were performed in triplicate.

### Endocrine Resistance Studies

For endocrine resistance studies, breast cancer cells were cultured in phenol-red free medium with 5% FBS for 48 hours. Following starvation from 17-β estradiol, cells were treated with 17-β estradiol (10^−10^ M) alone or in combination with 4-OH-tamoxifen (10^−7^ M) for 48 hours. Results are derived from three independent experiments (+/− s.d.). Cell cycle profile was performed by FACS as earlier described [23]. For *In Vitro* real time cell proliferation assay we employed the xCELLigence technology (ACEA). Following 48 hours starvation from 17-β estradiol, cells were treated with Fulvestrant (50 nM) and/or Alisertib (50 nM). Results are derived from three independent experiments.

### Immunoblot and Lenti-Vector Expression Studies

Immunoblot and lenti-vector expression studies were performed as previously described [23]. Antibodies employed for the immunoblot analysis were the followings: Aurora-A and P∼SMAD5 (Cell Signaling Technology, Boston, MA, USA). The Dominant Negative (DN) Aurora-A vector was kindly provided by Dr. Lomberk (Mayo Clinic, Rochester, MN).

## Supporting Information

Figure S1 **Role Of Aurora-A Over-Expression In ERα Down-Regulation.** (**A**) Immunoblot analysis showing parental and MCF-7 cells engineered to over-express Aurora-A. (**B**) Immunofluorescence analysis showing that Aurora-A over-expression induces partial ERα down-regulation and SMAD5 nuclear phosphorylation in ERα+MCF-7 cells. ERα (Abcam, Cambridge, Massachusetts, USA) was labeled in green, p∼SMAD5 (Cell Signaling Technology, Boston, MA, USA) was labeled in red and nuclei were labeled in blue with DAPI. (**C**) Graph showing the percentage of cells expressing p∼SMAD5 and ERα in vMCF-7^SMAD5^ and parental cells. Experiments were performed in triplicate (+/− s.d.).
(TIF)
