# Supplementary figures and images for: Aurora-A Mitotic Kinase Induces Endocrine Resistance through Down-Regulation of ERα Expression in Initially ERα+ Breast Cancer Cells
Source: PLoS One. 2014 May 9;9(5):e96995. doi: 10.1371/journal.pone.0096995 (PMC4016211; doi:10.1371/journal.pone.0096995)

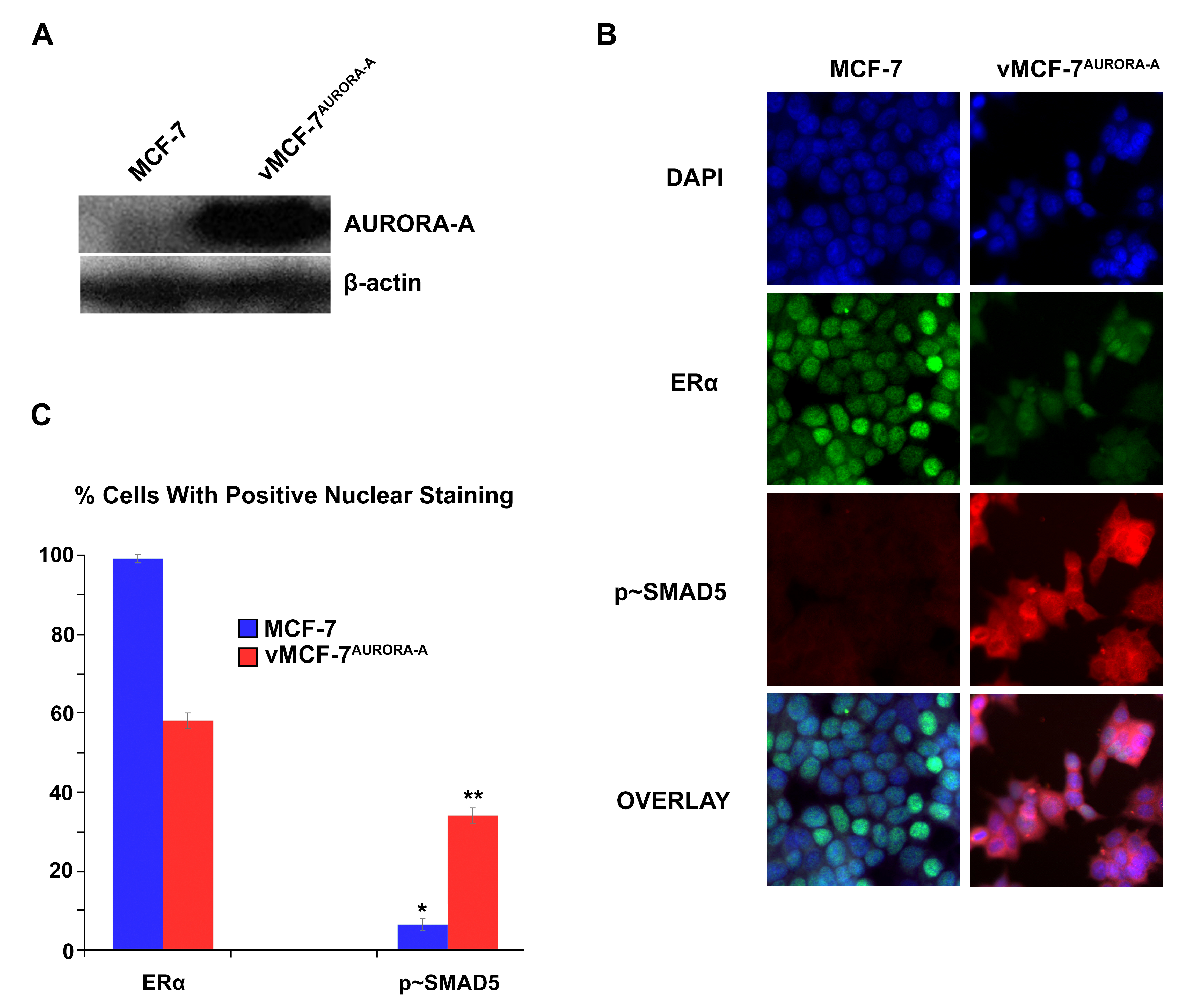

Supplement: Figure S1 — Role Of Aurora-A Over-Expression In ERα Down-Regulation. (A) Immunoblot analysis showing parental and MCF-7 cells engineered to over-express Aurora-A. (B) Immunofluorescence analysis showing that Aurora-A over-expression induces partial ERα down-regulation and SMAD5 nuclear phosphorylation in ERα+MCF-7 cells. ERα (Abcam, Cambridge, Massachusetts, USA) was labeled in green, p∼SMAD5 (Cell Signaling Technology, Boston, MA, USA) was labeled in red and nuclei were labeled in blue with DAPI. (C) Graph showing the percentage of cells expressing p∼SMAD5 and ERα in vMCF-7SMAD5 and parental cells. Experiments were performed in triplicate (+/− s.d.). (TIF) [file pone.0096995.s001.tif]
